# Commentary: Switching between internally and externally focused attention in obsessive-compulsive disorder: Abnormal visual cortex activation and connectivity

**DOI:** 10.3389/fpsyg.2017.01827

**Published:** 2017-10-16

**Authors:** John T. Vu, Gian R. Agtarap, Michael Wong

**Affiliations:** Bachelor of Health Sciences Program, McMaster University, Hamilton, ON, Canada

**Keywords:** obsessive-compulsive disorder, psychophysiology, visual cortex, attentional state, attentional switching, default mode network, task positive network, perceived responsibility

## Introduction

Obsessive Compulsive Disorder (OCD) is a debilitating psychiatric disorder characterized by intrusive, negative thoughts (obsessions) and repetitive behaviors (compulsions).

A puzzling feature of OCD is the inability of patients to utilize external information to terminate obsessional thinking. Recently, Stern et al. (2016) suggested this feature of OCD might stem from a difficulty in shifting from an internally focused attention state (i.e., obsessional thinking) to an externally focused one. The authors reported that subsequent to imagining a negative scenario (e.g., mother's cancer returns), OCD patients made more errors on an externally focused target detection task than did healthy control participants. This behavioral result coincided with hypoactivation of visual areas in OCD patients, which suggests that during obsessional thinking, patients may be less able to transition away from this internally focused state to process external visual information.

In this commentary, we expand on the study of Stern et al. (2016), discussing specifically how heterogeneity of OCD symptoms might have influenced their behavioral results. We then discuss how Stern et al.'s findings are interesting considering recent observations of an impaired visual system in OCD.

## Behavioral results

Stern et al. (2016) examined whether OCD patients' initial attention state would subsequently affect their performance on the target detection task. The experiment consisted of four experimental conditions, each one aimed at setting a particular initial attention state (prior to the target detection task): negative internally focused (by imagining an unpleasant event); positive internally focused (by imagining a pleasant event); externally focused (by performing a Stroop task); or neutral (by resting with their eyes opened). The results revealed that OCD patients made more errors on the target detection task, relative to control participants, if their initial attention state was negative internally focused. This led the authors to conclude that OCD patients have difficulty switching from an internally focused state to an externally focused one.

Given the authors' conclusion, the number of target detection task errors within the patient group should be greatest in the internally focused conditions; this, however, is not supported by Figure 2 (Stern et al., 2016). That is, although there was a trend for OCD patients to perform worse overall following a negative internally focused state, there is much overlap in the error bars between this condition and the others. It would be interesting to see if the difference in error rate between the negative internally focused condition and the others would increase if the negative imagined events were matched to the specific symptom profile of the patient. Hinds et al. (2012) showed that OCD patients responded, both behaviorally and physiologically, to external events in a symptom-specific manner. For example, after placing their hands in wet diapers, OCD patients with washing compulsions washed their hands longer and required more time for their physiology (as measured with Respiratory Sinus Arrhythmia; see Porges, 2007) to return to baseline than control participants. Interestingly, OCD patients with checking compulsions responded in a near-identical manner to control participants.

Similarly, studies have suggested the role perceived responsibility plays in OCD urges. When OCD patients were prompted to have high perceived responsibility over an imagined event (i.e., the patient is to blame for any negative outcome), they exhibited more OCD-like behaviors than participants who were prompted to have low perceived responsibility (i.e., the experimenter is to blame for any negative outcome) (Lopatka and Rachman, 1995; Arntz et al., 2007). As such, one might predict that error rate in the target detection task used by Stern et al. (2016) would increase as a function of the degree of perceived responsibility of the imagined event.

## Physiological results

To investigate the physiology of attentional switching in OCD, Stern et al. (2016) studied two specific neural networks that are reciprocally activated: the default mode network (DMN) and the task positive network (TPN) (see Fitzgerald et al., 2010; Stern et al., 2012). In an externally focused state, the TPN increases in activation while the DMN decreases in activation; the reverse is observed in an internally focused state. During the negative internally focused condition, Stern et al. observed hypoactivation of the TPN, specifically the left superior and bilateral inferior occipital cortex, among OCD patients. The authors hypothesized this was due to altered attention to visual stimuli during the target detection task, suggesting a difficulty switching from an internally focused state.

This finding is interesting in light of a hypothesis proposed by Gonçalves et al. (2010) that suggested OCD may be attributed to an impairment of visual processing. Anatomically, some studies have observed abnormal occipital lobe white matter levels among OCD patients (e.g., Fan et al., 2012). Other studies have found deficits in smooth-pursuit eye movements, indicating potential abnormalities in the frontal eye fields of OCD patients (Lencer et al., 2004). Moreover, when compared to control participants, OCD patients have been reported to experience difficulty in visually processing biological motion, which was attributed to impairment of the superior temporal sulcus (Kim et al., 2008). Although the target detection task, as used by Stern et al. (2016), did not test for either smooth-pursuit eye movements or visual processing of biological motion, further research may elucidate whether these visual deficits are related.

## Conclusion

The findings of Stern et al. (2016) have provided further insight into the psychophysiology of OCD. The physiological deficits in the networks involved in internal and external attention states may be reflected by the behavioral results seen in the attention switching task. Most notably seen was hypoactivation of the occipital cortex. As the authors suggested, this hypoactivation may increase the difficulty that OCD patients have with processing external visual information. It would be interesting to investigate whether similar impairments are observed in other sensory systems, such as audition or touch, as sources of external evidence are arguably not restricted to vision. For instance, locking a door is a multisensory process, involving at least vision, audition, and touch; if OCD is explained solely by an impaired visual system, then what role, if any, do the other sensory systems play in providing a *safety* cue?

## Author contributions

All authors listed have made a substantial, direct and intellectual contribution to the work, and approved it for publication.

### Conflict of interest statement

The authors declare that the research was conducted in the absence of any commercial or financial relationships that could be construed as a potential conflict of interest.
